# Shifts in ovine cardiopulmonary microRNA expression in late gestation and the perinatal period

**DOI:** 10.1371/journal.pone.0204038

**Published:** 2018-09-19

**Authors:** Ramona H. Krauss, Belinda Phipson, Alicia Oshlack, Nikita Prasad-Gupta, Michael M. Cheung, Joseph J. Smolich, Salvatore Pepe

**Affiliations:** 1 Heart Research, Murdoch Children’s Research Institute, Melbourne, Australia; 2 Department of Paediatrics, University of Melbourne, Melbourne, Australia; 3 Bioinformatics, Murdoch Children’s Research Institute, Melbourne, Australia; 4 Department of Cardiology, Royal Children’s Hospital, Melbourne, Australia; University of South Alabama Mitchell Cancer Institute, UNITED STATES

## Abstract

**Background:**

MicroRNAs (miRNAs) have been identified as important contributors to the regulation of early fetal cardiopulmonary development. However, miRNA expression profiles during late gestation and the early neonatal period are not fully elaborated in large mammals such as sheep (*ovis aries*). The aim of this study was to sequence miRNA from cardiopulmonary tissues in late gestation and neonate sheep to identify changes in miRNA expression.

**Methods:**

Illumina HiSeq next-generation deep sequencing (NGS) was performed on ovine tissues from the left (LV) and right ventricles (RV), lungs and pulmonary artery (PA) of preterm fetuses (128 days), near-term fetuses (140 days) (term = 148 days) and neonatal lambs (5 days). NGS reads were mapped to the sheep genome (*OviAri)* and published miRNA sequences.

**Results:**

Of 1345 cardiopulmonary miRNAs that were sequenced, relatively few major shifts in miRNA expression were detected with increased age from near term to neonates, and were confirmed by quantitative real-time PCR: bta-miR-146a (lung), bta-miR-22-3p (lung, LV), hsa-miR-335* (lung, PA), and miR-210 (lung, PA, LV).

**Conclusions:**

Sequencing of miRNA led to identification of four predominant miRNA in ovine cardiopulmonary tissues which alter expression during late gestation and the early neonatal period, concurrent with important functional changes in heart and lungs.

## Introduction

MicroRNAs (miRNA) are short noncoding RNAs (18–25 nucleotides) that exert intricate regulation of cellular processes by post-transcriptional inhibition of specific gene expression. Via incorporation in the RNA-induced silencing complex (RISC), miRNAs repress RNA translation or promote mRNA degradation, preventing protein translation [1]. A critical role for miRNAs in mammalian cardiac development has been identified by studies in mice with cardiac-specific deficits of mature miRNAs that disrupt heart morphogenesis in early gestation [2–4]. In addition, upregulation of the miR-15 family has been implicated in cardiomyocyte binucleation and cessation of cardiomyocyte proliferation after birth [5], while a postnatal switch from the slow (fetal) β-myosin heavy chain to the faster (mature) α–myosin heavy chain in cardiomyocytes has been linked to expression shifts in the miR-208 family [6]. Furthermore, a role for miRNAs in regulating lung development has been identified in rats [7–9], and mice [10–12]. However, in part due to their short gestation, many temporal aspects of heart and lung development in rodents differ from those of humans. For example, rats and mice have no detectable alveoli in the lungs at birth, while development of the coronary circulation in rats is not completed until after birth [13–15].

By contrast to rodents, the fetal and newborn cardiopulmonary physiology of sheep (*ovis aries*) shares similar important features with humans [16,17]. However, apart from a recent microarray study of left ventricular (LV) microRNA expression in fetal, neonatal and juvenile sheep [18], there is a dearth of large animal studies examining changes in cardiac and pulmonary miRNA expression between late-gestation and the early neonatal period. Altered expression patterns of miRNA are anticipated with key changes between the late gestation and neonatal period such as: structural maturation of the fluid-filled lungs with emergence of surfactant production; a shift from right ventricular (RV) to LV functional dominance and augmentation of cardiac pumping performance; a marked fall in pulmonary arterial blood pressures; and the dramatic switch to air-breathing lungs at birth [16,17,19,20].

Accordingly, the aim of this study was to determine the late gestation changes in miRNA expression occurring between preterm fetal, near-term fetal and neonatal sheep (*ovis aries*) in LV and RV myocardium, main pulmonary artery (PA) and lungs. Due to the few ovine miRNA entries available in databases such as miRBase [21], and the sizable limitations of microarray profiling [22], which include insensitivity to detect single nucleotide differences between species (thus limiting the capacity to detect a greater range of miRNA and previously unidentified miRNAs), we therefore used next-generation deep sequencing (NGS) to expand the cardiopulmonary sheep ‘miRNome’. Validation of shifts in expression of specific miRNAs was performed by quantitative real time RT-PCR (qRT-PCR) using specific primers (TAQMAN and custom designed) for both reverse transcription and qRT-PCR.

## Methods

### Animals

All animal study protocols were approved by the Murdoch Children’s Research Institute Animal Ethics Committee. Border-Leicester cross ewes with documented time-monitored mating and pregnancies were purchased from a licensed supplier of sheep for scientific use (Victoria, Australia). Sheep welfare, husbandry, accommodation, nutrition, transport and experimental protocols were managed and performed according to the Australian Code of Practice for the Care and Use of Animals for Scientific Purposes (National Health & Medical Research Council of Australia, 8th edition). After transportation to the research facility, prior to experimental use, ewes were acclimatised for 1–3 weeks under veterinary surveillance and daily monitoring. Sheep were penned individually but roomed with companions and conditioned to staff and noise. Neonates were penned with their mother. Feed intake was divided into two portions. In addition, lucerne hay was provided in hanging baskets to allow time for active foraging for food. Cage floors were covered with rubber mats to ensure comfort of footing.

For the collection of fetal samples, pregnant Border-Leicester cross ewes at a gestation of either 127–129 days (PT group) or 140–142 days (NT group) were anaesthetized with an intramuscular injection of ketamine (5mg/kg) and xylazine (0.1mg/kg), followed by inhalation of isoflurane (5%) delivered by mask [23]. After tracheal intubation, anaesthesia was maintained with 2–3% isoflurane and nitrous oxide (10–20%) in oxygen-enriched air delivered via a volume-controlled ventilator (900C Servo; Siemens-Elema, Solna, Sweden), supplemented by intravenous infusion of ketamine (1–1.5mg/kg/h), midazolam (0.1–0.15mg/kg/h), and fentanyl (2–2.5mg/kg/h). Fetuses were delivered via a midline laparotomy and hysterotomy and euthanized with an overdose of sodium pentobarbitone (100 mg/kg) prior to collection of tissue samples. Ewes and neonatal lambs (Neo, 5–7 days of age) were euthanized with an overdose of sodium pentobarbitone.

### Processing of tissue samples and RNA extraction

Fetal and neonatal tissues were dissected on ice immediately after euthanasia, with samples collected from the LV and RV free walls of the heart, the main PA arising from the RV, and the right (mid-lobe) lung. All tissues samples were snap frozen and stored at -80°C after dissection.

Frozen tissue samples (50 mg) were ground into a fine powder using a mortar and pestle immersed in liquid nitrogen. Total RNA extractions from tissues were performed with the miRNeasy kit from Qiagen (catalog number 217004) according to the manufacturer’s recommendations with the following modifications:

1) the incubation in Qiazol (step 4) was extended to 8 minutes to improve RNA quality; and 2) the mixing time with chloroform (step 5) was extended to 30 seconds. For Illumina HiSeq next-generation deep sequencing (NGS) samples, the optional on column DNase digestion was performed using the DNase kit from Qiagen (catalog number 79254) according to the manufacturer’s protocol after step 10. Total RNA was eluted in 35μl Nuclease-free water in the last step.

### Illumina HiSeq next-generation deep sequencing

Illumina HiSeq next-generation deep sequencing (NGS) was performed by the Australian Genome Research Facility platform in the Walter and Eliza Hall Institute of Medical Research in Melbourne, Australia. The TruSeq Small RNA Library Preparation Kit with a size selection range of 145–160 nt (RNA selection 18–33 nt) was used for library preparation. The standard protocol was run for 50 cycles.

#### Pre-processing and analysis of miRNA-Seq data

The first data processing step involved adaptor trimming and size selection using the cutadapt tool [24]. First, N’s were removed from the 5’ end of the sequences, and then the 5’ and 3’ adaptor sequences and their reverse complements were removed sequentially. In the final step, all sequences outside the 15-32nt range, the common length for miRNAs, were excluded.

Next, NGS reads were mapped to the sheep genome, version OviAri3, from the UCSC website (http://genome.ucsc.edu/). The bowtie aligner, which is designed for short reads, was used for mapping the reads [25]. As the sheep reference genome may not be complete and sheep are heterozygous with respect to genetic background, up to two mismatches were allowed, and the “best” mapped reads were retained.

The next step was to annotate the miRNAs and exclude small RNA species other than miRNAs from the analysis. As only a limited number of miRNAs have been cloned in sheep, all miRNAs listed online in miRBase, version 21 (http://mirbase.org/), as well as recently published sheep miRNAs [26–28] were used for annotating the miRNAs as follows. The sequences of all the previously published sheep miRNAs, as well as known miRNAs across all species in miRBase were mapped to the sheep genome, after converting U’s to T’s with the FASTX-Toolkit (http://hannonlab.cshl.edu/fastx_toolkit/). The alignment was performed using bowtie, specifying no mismatches and retaining the best alignment. Redundant miRNAs were removed from the combined list such that all miRNAs are non-overlapping using a custom R script. The result was a custom annotation file with a list of 2388 miRNAs mapping to *OvisAri3*, with chromosomal location for each miRNA. This was used for quantification of the miRNAs in the sheep samples.

**Fig 1:**
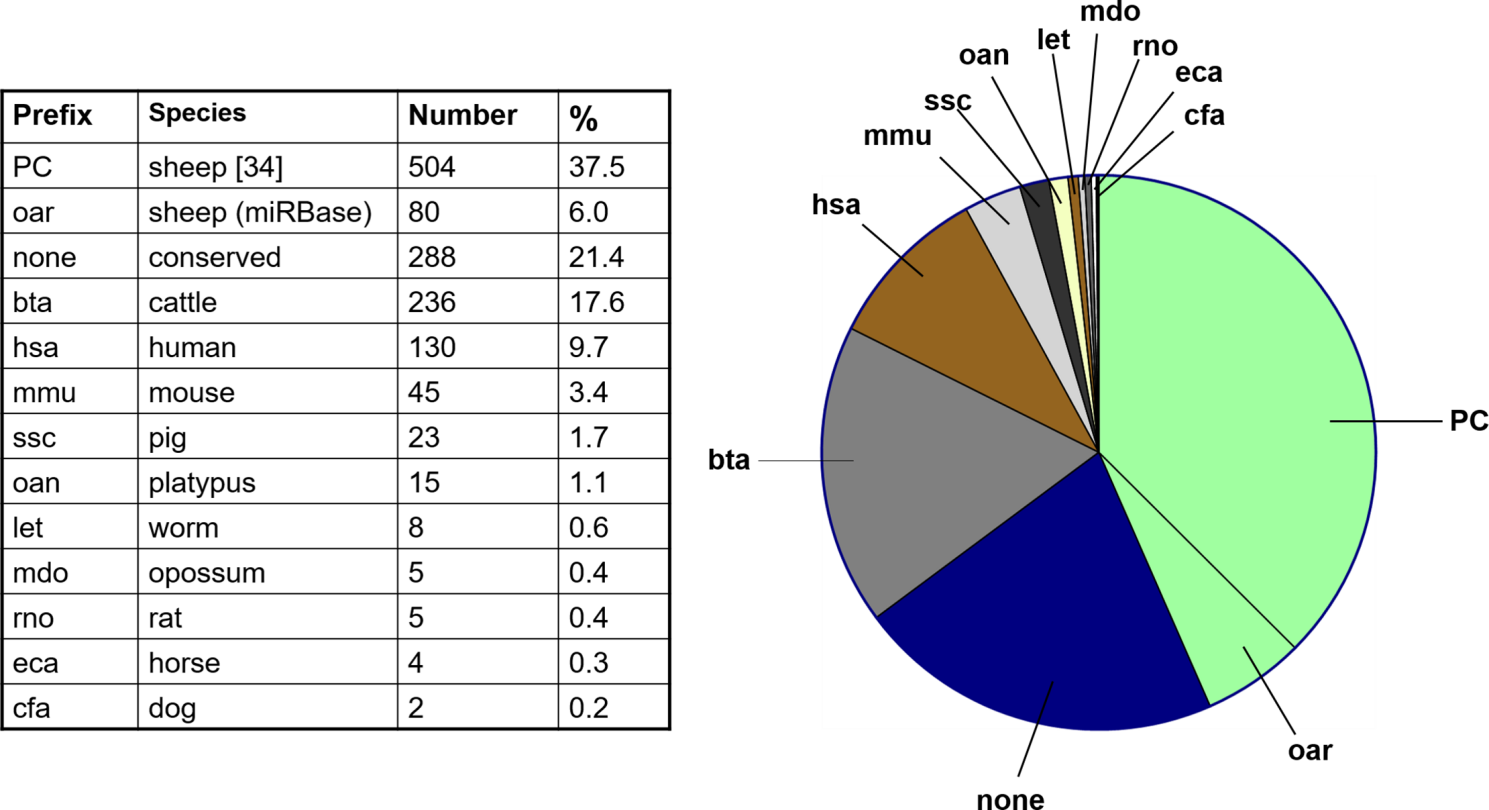
The proportion of miRNA reads detected in Illumina HiSeq next-generation deep sequencing (NGS) which mapped to the sheep genome version *OviAri3* as well as recently identified ovine miRNAs (‘PC’ [34]) or miRNAs of the miRBase (http://mirbase.org/). The miRNA prefix defines the species in which the identified miRNA was cloned: bta = bos taurus (cattle), hsa = homo sapiens (human), oar = ovis aries (sheep), mmu = mus musculus (mouse), ssc = sus scrofa (pig), oan = ornithorhynchus anatinus (platypus), let = caenorhabditis elegans (worm), mdo = monodelphis domestica (opossum), rno = rattus norvegicus (rat), eca = equus caballus (horse), cfa = canis familiaris (dog); none = no species-specific prefix (conserved sequence across species). Number: number of miRNAs mapping to sequences of a specific species. 5: percentage of total detected miRNAs. NB: All identified miRNAs are listed in **S1 Table**.

For statistical analysis, only miRNAs that mapped to the sheep genome and overlapped with the custom annotated miRNAs were included. Reads were counted over the annotated miRNAs using the *featureCounts* software [29]. Lowly expressed miRNAs were filtered out by retaining only those miRNAs that had at least one count per million in at least three samples. Thus, a list of 1345 miRNAs that are expressed in at least one condition and with the ‘best’ alignment was created, which we refer to as the ‘cardiopulmonary sheep miRNome’ in the text. All miRNAs of the ‘cardiopulmonary sheep miRNome’ including their chromosomal location, orientation and sequences are listed in S1 Table. Detailed information about miRNA nomenclature is reported by Griffith-Jones, et al, [30] as well as in the miRBase, and as cited above [26–28]. Species-specific prefixes are detailed in Fig 1.

The raw counts were analysed in the R statistical computing environment, using the edgeR [25] and limma [31] Bioconductor [32] packages. The data was TMM normalized [24] to account for composition bias; and cyclic loess normalization applied in the voom transformation step [27]. Differential expression for the comparisons of interest was assessed using moderated t-statistics in each tissue separately, using the ‘robust = TRUE’ option to account for outliers [33]. miRNAs were called significantly differentially expressed if the Benjamini and Hochberg adjusted false discovery rates [34] were less than 15%. Only miRNAs with an AveLogExp>1 were included for further analysis.

#### Reverse Transcription and qRT-PCR

The expression of identified miRNAs was quantified using the TaqMan miRNA expression assays from Applied Biosystems (AB) listed in Table 1.

**Table 1:** TaqMan Assays used for qRT-PCR measurements. miRNA-ID: miRNA ID: microRNA name as listed in S1 Table. Assay name and catalog #: commercially available assay from Applied Biosystems. Sequence: sequence of the miRNA profiled by TaqMan assay.

miRNA-ID	Sequence	Assay name	catalog #
bta-miR-22-3p_R+1	AAGCUGCCAGUUGAAGAACUG	bta-miR-22-3p	4440886
bta-miR-27a-3p_R+1	UUCACAGUGGCUAAGUUCCGC	hsa-miR-27a	4427975
bta-miR-146a_R-2	UGAGAACUGAAUUCCAUAGGUU	xtr-miR-146	4440886
miR-210	CUGUGCGUGUGACAGCGGCUG	custom designed	4398987
hsa-miR-335*	UUUUUCAUUAUUGCUCCUGACC	hsa-miR-335*	4427975
PC-5p-592_3212	UAGCAGCACGUAAAUAUUGGG	custom designed	4398987
bta-miR-2284x_R+1	UGAAaaGUUCGUUCGGGUUUU	bta-miR-2284x	4440886

Total extracted RNA (10ng/sample) underwent Reverse Transcription to produce miRNA-specific cDNA using the TaqMan miRNA Reverse Transcription kit (Applied Biosystems, catalog number 4366597) according to the manufacturer’s protocol. cDNA samples were stored at -30°C until required. Per well, 1.34μl Reverse Transcription product was mixed with 7.66μl Nuclease-free water and 10μl TaqMan 2X Universal PCR Master Mix, NoAmpErase UNG (Applied Biosystems, catalog number 4364341). All reactions were run in triplicates except where specified otherwise in a 7900HT Fast Real-Timer PCR System (Applied Biosystems) according to the manufacturer’s recommendations.

Fold changes were calculated with the common ΔΔCt method described by Livak and Schmittgen [35]. Ct values were normalized to the average of two miRNAs which showed the lowest SD across all tissue samples in the NGS analysis. Thus, ΔCt values were calculated as follows:

ΔCt=Ct(testedmiRNA)‑averageCt(bta‑miR‑2284x,PC‑5p‑592_3212).

For the comparisons of miRNA expression between the age groups in each cardiopulmonary tissue ΔΔCt values were calculated as follows:

Comparison1)Near‑termversuspretermfetuses:ΔΔCt=ΔCt(NT)‑ΔCt(PT)

Comparison2)Neonateversusnear‑termfetuses:ΔΔCt=ΔCt(Neo)‑ΔCt(NT)

Fold changes were calculated as the relationship 2^-ΔΔCt^.

ΔCt values were compared in statistical analyses using GraphPad Prism 6. Data were calculated using one-way analysis of variance (ANOVA) followed by unpaired Student's tests, with p values adjusted with a Bonferroni correction for multiple comparisons.

## Results

### Identification of ovine miRNAs by NGS

To create a novel ‘cardiopulmonary sheep miRNome’, Illumina HiSeq NGS was performed on tissue samples collected from lung, PA, RV and LV of three different developmental age groups (n = 3 per group): preterm fetuses (PT) 128 days in gestation (term = 147 days), near-term fetuses (NT) 140 days in gestation, and neonatal lambs (Neo) aged 5–7 days. NGS reads detected across all samples were mapped to the sheep genome version *OviAri3* and annotated with miRNAs across all species from miRBase, including more recently published ovine miRNAs [26].

Across all tested tissue samples (lung, PA, LV and RV), 1345 miRNAs were annotated using a novel bioinformatics strategy (see Methods section for details). We refer to these 1345 miRNAs as the ‘cardiopulmonary sheep miRNome’, with details of all miRNAs, including their chromosomal locations and sequences listed in S1 Table. A prefix defines the species in which the miRNA has been cloned, whereas miRNAs without a prefix are conserved miRNAs with identical sequences across species.

Fig 1 summarizes the overlap of the detected miRNAs with miRNA sequences published in *ovis aries* and other species. Of the 1345 detected miRNAs, 584 were sheep-specific miRNAs, with only 80 listed as ovine miRNAs (prefix = oar) in the miRBase, and 504 being more recently published miRNAs (prefix = PC) [26]. A high number (288) of the detected miRNAs were non-specific cross-species conserved miRNAs (no miRNA prefix). A similarly high number (236) of detected miRNAs were cattle-specific miRNAs (prefix = bta). Notably 130 detected miRNAs were identified as human-specific miRNAs (prefix = hsa). The remaining 62 miRNAs mapped to miRNA sequences previously identified for other species including mouse, rat and zebrafish. Considering the high proportion of gene homology (>90%) between sheep, cattle and human lungs, the 584 sheep-specific miRNA are likely variants that differ to bovine or human by one or a few base-pairs.

### Age-group comparisons of miRNA expression measured by NGS

To identify changes in miRNA expression occurring during ovine cardiopulmonary development, differential expression of the miRNAs between the different age groups was assessed in each tissue separately (n = 3 per age group). The miRNA expression changes were compared between PT and NT, and between NT and Neo groups. Differentially expressed miRNAs were detected using moderated t-statistics in the limma package [31]. The results for all miRNAs of the ‘cardiopulmonary sheep miRNome’ in each tissue are listed in S1 Table.

MicroRNAs were further investigated if they had an average log expression (AveLogExp) of >1 count per million (cpm) and their fold changes achieved a false discovery rate (FDR) of <15% (see ‘Methods’ for further details of analysis). Under these criteria, 84 miRNA expression changes were detected across all tissues (41 decreases and 43 increases). Of these, 78 occurred from NT to Neo, and only six from PT to NT, with the majority of changes in miRNA expression (59) detected in the lung.

Comparing changes between PT and NT groups, expression of only one miRNA (PC-3p-30485_40) decreased in the lung. On the other hand, in the LV, three miRNAs were up-regulated (miR-7641, miR-3074-5p and bta-miR-221_R+1) and two down-regulated (bta-miR-148a, oar-miR-370-3p_R-2). By contrast, no miRNAs changed expression in the RV and PA between the PT and NT groups at FDR<0.15 and AveLogExp>1.

Comparing miRNA expression between NT and Neo at FDR<0.15 and AveLogExp>1, 58 miRNAs changed expression in the lung (32 were up-regulated and 26 down-regulated), 16 in the PA (six up-regulated, 10 down-regulated), four in the LV (two up-regulated and two down-regulated) and none in the RV (see Table 2). Notably, two of the largest changes included a 20-fold increase in LV for mmu-miR-208a-5p and a 20-fold increase in PA for PC-5p-874723_1. Changes in miRNA expression between NT fetuses and Neo lambs in the lung included bta-miR-15b (39% increase, FDR = 0.11), hsa-miR-24-2*_L+1R-1 (93% increase, FDR = 0.07), miR-29a (83% increase, FDR = 0.15), bta-miR-27a-3p_R+1 (70% increase, FDR: 0.02) and bta-miR-146a_R-2 (168% increase, FDR = 0.04).

**Table 2:** Near-term (NT) versus neonate (Neo) comparison of changes in miRNA to FDR<0.15. miRNA ID: microRNA name as listed in Supplementary Data 1. AvgLogExp: average log expression, cpm: counts per million, FC: fold change, FDR: false discovery rate, PA: pulmonary artery, LV: left ventricle, RV: right ventricle, n.d.: no difference detected. (n = 3), NB: data is sorted by FDR.

Tissue	miRNA ID	AvgLogExp (cpm)	FC	FDR
**Lung**	bta-miR-22-3p_R+1	13.670	2.041	0.022
	bta-miR-27a-3p_R+1	10.604	1.700	0.022
	miR-210	6.461	0.476	0.022
	bta-miR-92a_R+1	13.596	0.644	0.031
	hsa-miR-335*	7.163	2.050	0.031
	miR-668-3p	2.997	0.388	0.031
	hsa-miR-4448_R-3_1ss6CG	1.806	4.750	0.031
	bta-miR-181a_R-1	14.572	0.701	0.034
	miR-181a	14.570	0.697	0.034
	miR-92a-3p	13.138	0.645	0.040
	hsa-miR-125b-1*	4.604	2.279	0.040
	bta-let-7a-2-p3	12.368	1.285	0.042
	bta-miR-146a_R-2	6.930	2.683	0.042
	miR-142-3p	4.924	2.738	0.045
	bta-miR-504_R+1	5.318	0.482	0.049
	PC-3p-30485_40	4.459	0.581	0.055
	bta-miR-146b	4.837	3.743	0.056
	miR-133	4.962	0.644	0.064
	let-7	12.739	1.254	0.069
	bta-miR-23a	9.264	1.491	0.069
	miR-181a-2-3p	9.014	0.617	0.069
	bta-miR-21_R-1	2.287	2.546	0.069
	miR-142b	9.234	1.981	0.070
	bta-miR-378	9.099	1.536	0.070
	miR-222-3p	7.495	1.614	0.070
	bta-miR-328	5.540	0.573	0.070
	miR-148b-5p	5.242	1.606	0.070
	bta-miR-223	4.582	4.969	0.070
	miR-1306-5p	3.701	0.595	0.070
	hsa-miR-24-2*_L+1R-1	4.957	1.928	0.071
	bta-miR-191_R-1	13.630	1.547	0.076
	miR-144-3p	4.981	2.021	0.076
	miR-323	3.089	0.452	0.076
	mmu-miR-503*_L+1R-3_1ss15AG	3.448	0.49	0.085
	bta-miR-25	12.341	0.849	0.090
	hsa-let-7d*	7.096	0.608	0.090
	bta-miR-1343*_L+1R+2	5.993	0.682	0.090
	oar-miR-485-5p	5.616	0.657	0.100
	bta-miR-30e-5p	11.853	1.47	0.113
	miR-192	10.965	1.246	0.113
	bta-miR-1468	10.679	1.76	0.113
	bta-miR-148b	9.902	1.275	0.113
	miR-24	8.570	1.423	0.113
	bta-miR-15b	7.059	1.393	0.113
	miR-155	6.703	1.409	0.113
	bta-miR-424*	6.309	0.668	0.113
	miR-1260	3.521	2.076	0.113
	bta-miR-96	2.639	0.372	0.113
	miR-223-5p	1.765	4.44	0.113
	oar-miR-323c	5.590	0.563	0.114
	bta-miR-2483*	5.120	0.557	0.123
	miR-127	15.017	0.769	0.143
	bta-miR-130a	10.981	0.806	0.143
	oar-miR-410-3p	9.152	0.7	0.143
	bta-miR-363_L+1	4.824	0.572	0.143
	miR-3074-5p	9.780	1.386	0.144
	bta-miR-7	2.342	1.673	0.144
	miR-29a	6.802	1.826	0.149
**PA**	bta-miR-10a_R-1	14.450	0.221	3E-04
	PC-5p-874723_1	2.760	20.32	3E-04
	bta-miR-98	11.250	2.025	0.013
	bta-miR-490_R+1	3.492	0.2	0.018
	hsa-miR-148a*_L+1	8.203	1.887	0.049
	hsa-miR-335*	7.915	0.422	0.049
	miR-499b-3p	4.513	0.199	0.049
	bta-miR-22-3p_R+1	15.100	1.534	0.051
	miR-1	5.549	0.471	0.063
	hsa-miR-10a*_R-1	3.254	0.192	0.063
	miR-210	8.370	0.444	0.073
	bta-miR-143_R-1	19.100	0.449	0.102
	miR-181a	13.170	0.627	0.113
	bta-miR-181a_R-1	13.170	0.629	0.113
	miR-409b	9.996	1.768	0.113
	miR-296-3p	6.497	1.932	0.113
**LV**	bta-miR-22-3p_R+1	14.832	2.008	0.058
	bta-miR-424	8.196	0.557	0.118
	mmu-miR-208a-5p	2.293	20.47	0.118
	miR-210	5.913	0.479	0.127
**RV**	n.d.			

It is noteworthy that, between the NT and Neo groups, altered expression of three miRNA occurred in more than one tissue. These comprised: *1)* bta-miR-22-3p_R+1, which increased in the lung (by 100%, FDR = 0.02), PA (by 53%, FDR = 0.05) and LV (by 100%, FDR = 0.06): *2)* miR-210 which decreased in the lung (by 52%, FDR = 0.02), PA (by 56%, FDR = 0.07) and LV (by 52%, FDR = 0.13) and; *3)* hsa-miR-335* which increased in the lung (by 100%, FDR = 0.03) but decreased in the PA (by 58%, FDR = 0.05).

### Age-group comparisons of miRNA expression validated by qRT-PCR in a second series of sheep

Five miRNA candidates were selected from the NGS study for validation in a second independent series of PT, NT and Neo sheep by qRT-PCR (n = 4 per group), namely bta-miR-22-3p_R+1, bta-miR-27a-3p_R+1, bta-miR-146a_R-2, miR-210 and hsa-miR-335*.

**Fig 2:**
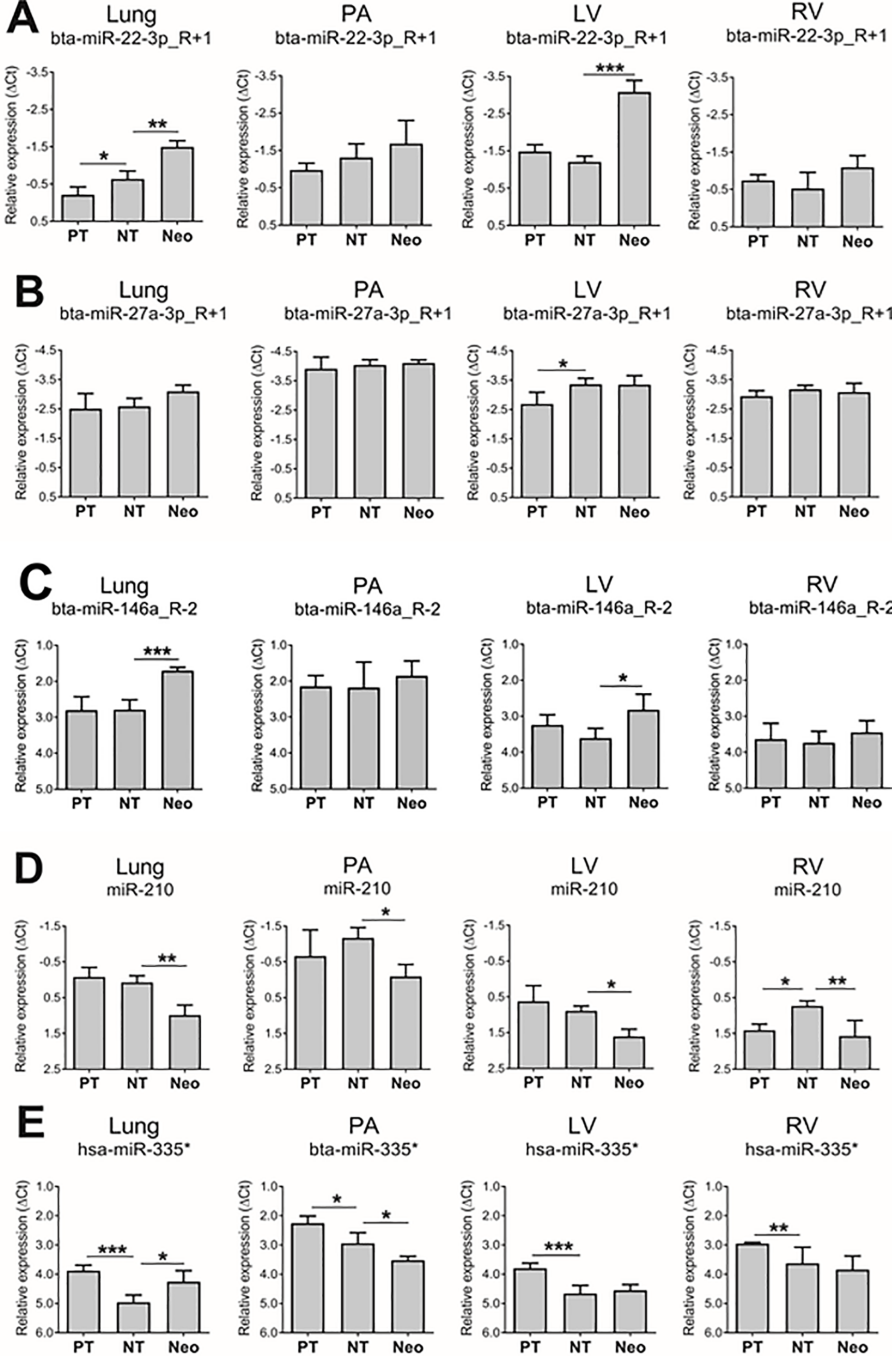
The expression patterns of miRNA confirmed by qRT-PCR in lung, pulmonary artery (PA), left ventricle (LV), and right ventricle (RV) from three age groups: preterm fetus (PT), near-term fetus (NT), neonatal lamb (Neo). **A.** bta-miR-22-3p_R+1, **B.** bta-miR-27a_R+1. **C.** bta-miR-146a_R-2, **D.** miR-210, **E.** hsa-miR-335*. *p<0.05, **p<0.01, ***p<0.001. Data are represented as mean±SD, n = 4.

As shown in Fig 2A, bta-miR-22-3p_R+1 expression increased in the lung from PT to NT (by 50%, p<0.05) and from NT to Neo (by 82%, p<0.01), and markedly in the LV from NT to Neo (by 267%, p<0.001). However, bta-miR-27a-3p_R+1 expression (Fig 2B) increased in the LV between PT and NT (by 59%, p<0.05), but was unchanged in tested tissues between NT and Neo. By contrast, Bta-miR-146a_R-2 expression (Fig 2C) was unaltered in tested tissues from PT to NT, but between NT and Neo, increased in the lung (by 112%, p<0.001) and LV (by 72%, p<0.01). On the other hand, miR-210 expression (Fig 2D) increased from PT to NT in the RV (by 60%, p<0.05), but between NT and Neo, decreased in lung (by 47%, p<0.01), PA (by 53%, p<0.05), LV (by 39%, p<0.05) and RV (by 44%, p<0.01). Hsa-miR-335* expression (Fig 2E) decreased from PT to NT in the lung (by 53%, p<0.001), PA (by 38%, p<0.05), LV (by 27%, p<0.001) and RV (by 37%, p<0.01), and between NT and Neo, decreased in the PA (by 33%, p<0.05), but increased in the lung (by 62%, p<0.05).

### Comparison of the results in sheep from the NGS and qRT-PCR studies

All age-related miRNA expression changes detected at FDR<0.15 and AveLogExp>1 in the NGS study were confirmed at p<0.05 in the qRT-PCR study, except for the apparent increased expression of bta-miR-22-3p_R+1 in the PA and bta-miR-27a-3p_R+1 in the lung, which did not validate by qRT-PCR (p = 0.50 and p = 0.15 respectively).

When profiling miRNA expression in the qRT-PCR validation group, ten additional significant miRNA expression changes were detected that were not evident at FDR<0.15 by NGS. Between PT and NT, these comprised three additional increases (bta-miR-22-3p_R+1 in the lung, bta-miR-27a-3p_R+1 in the LV and miR-210 the RV) and four decreases (hsa-miR-335* in the lung, PA, LV and RV). The corresponding changes from NT to Neo were two increases (bta-miR-22-3p_R+1 in the RV and bta-miR-146a_R-2 in the LV) and one decrease (miR-210 in the RV). Of note, all additional significant age-related changes detected with qRT-PCR showed fold-changes in the same direction as in the NGS analysis, however at FDR>0.15 (S1 Table).

**Fig 3:**
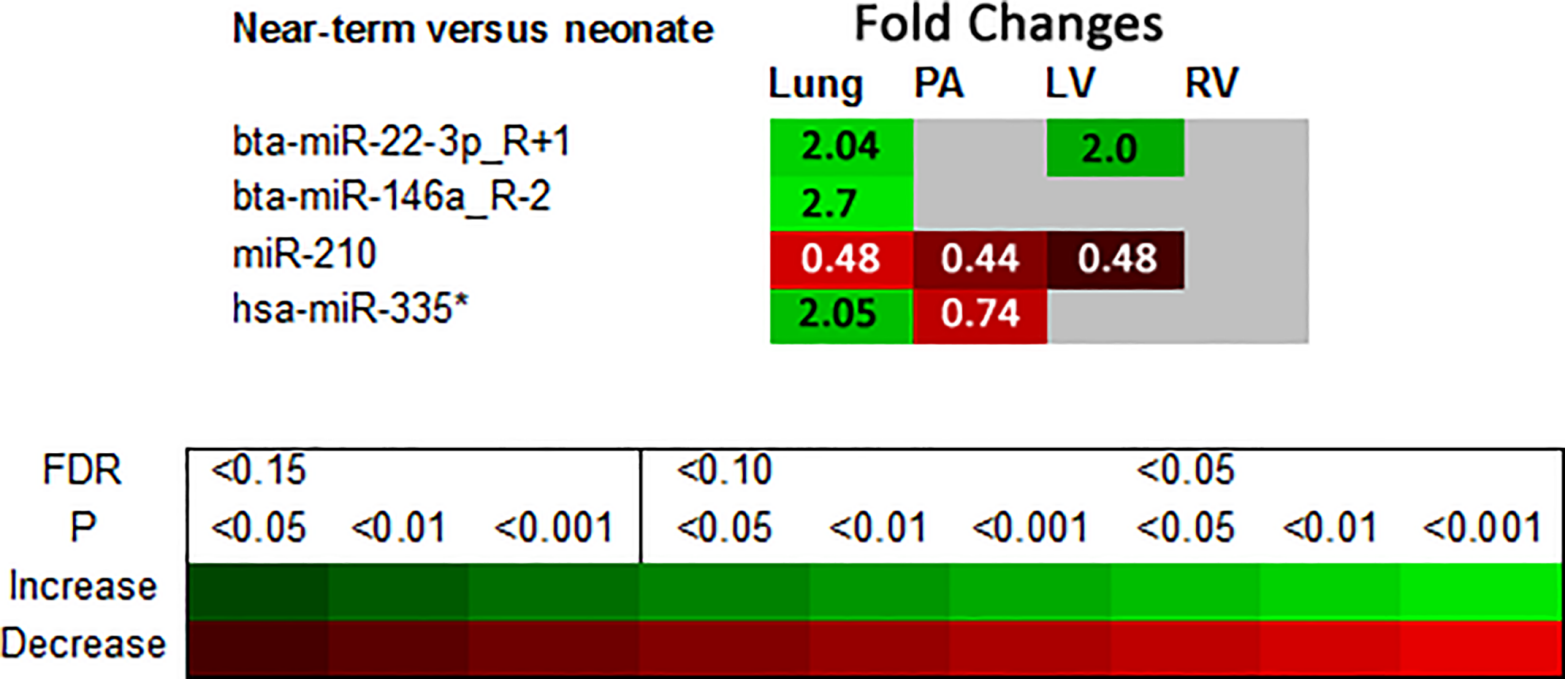
The miRNA expression fold changes which were confirmed in both the sheep group used for NGS (FDR<0.15) and the sheep group used for qRT-PCR (p<0.05) are summarised for changes between near-term fetuses and neonatal lambs. Fold Change increases in expression are green and decreases red. Legend color variations indicate significance levels. FDR = false discovery rate for Illumina HiSeq next-generation deep sequencing (NGS) and P = adjusted p-value for quantitative real-time PCR (qRT-PCR), n = 7 (NGS = 3; qRT-PCR = 4).

Fig 3 summarizes fold changes in miRNA expression confirmed at both FDR<0.15 by NGS and p<0.05 by qRT-PCR, and indicates that changes significant with both techniques were present from NT to Neo, but not between PT and NT. These changes were evident in bta-miR-22-3p_R+1 (increase in lung and LV), miR-210 (decrease in lung, PA and LV), hsa-miR-335* (increase in lung and decrease in PA) and bta-miR-146a_R-2 (increase in lung).

## Discussion

Our study has identified a total of 1345 miRNAs in fetal and neonatal ovine cardiopulmonary tissues. With only 584 miRNA identified as sheep specific, only 80 of these miRNAs are listed in the miRBase as sheep-specific miRNAs (miRBAse-OAR3.1). Although this reinforces the need to further identify and consolidate the sheep miRNA expression during fetal and neonatal development of the heart and lungs. Most ovine miRNAs published in the miRBase have been cloned in ovine skeletal muscle, whereas organ and region-specific or cell type-specific expression differences in miRNA nucleotide sequences have had very limited detailed study. Thus it is not surprising that only a small number of the miRNAs detected in our study mapped to ovine miRNAs listed in the miRBase. Many of the 584 sheep-specific miRNA vary by as little as a single base pair to human or other species. The finding that a high number of miRNAs detected in our study had conserved sequences common to other species justifies the integration of miRNAs which mapped to sequences of other species in the analysis. In particular, there was a predominant overlap of the identified ovine miRNA sequences with bovine-specific miRNA sequences. More work is required to determine and validate whether such minor base pair differences reflect only species-specific genetic and protein differences or potentially differences in miRNA function, including cell type-specific fine regulatory function.

Only relatively few of the 1345 miRNAs detected by NGS in the ovine cardiopulmonary tissues showed differential expression that progressed according to age, suggesting that there may be strict temporal and spatial regulation of miRNA expression during cardiopulmonary development in late gestation and the early postnatal period. Although tissue fragmentation methods (mechanical or enzymatic) for subsequent RNA extraction are known to produce a distribution of fragment sizes, it is highly likely that tissue type may also influence fragment size distribution on the basis of tissue density and fibrous composition. As the library sizes for the LV and RV samples were smaller than the library sizes for the PA and lung samples, this may have had some influence on NGS sensitivity to detect miRNA expression changes in LV and RV. A high variation in library sizes across different samples commonly occurs when performing NGS [36]. It is unclear whether the differences in library sizes occurring in our study represent a true biological variation as a potential limitation in the detection of significant age-related changes was the small sample size of the NGS series (n = 3).

When measuring miRNA expression by qRT-PCR, more miRNA expression changes between the age groups could be detected compared to NGS. The reason for the discrepancy of the results may relate to differing experimental and statistical approaches of NGS and qRT-PCR along with limited subject numbers. Most miRNAs shifts were found to change in more than one tissue, indicative of roles at multiple targets. Although some of these miRNA have previously been reported for murine heart, their functional role and targets have not been determined in the context of fetal and neonatal ovine cardiopulmonary development.

It is important to consider miRNA expression in the context of the marked changes in ovine cardiopulmonary physiology that occur during the latter part of gestation, and more particularly, between the fetal and newborn periods [16,17,19,20,37]. The fetal circulation has an “in parallel” and compartmentalized organization whereby both ventricles contribute to perfusion of fetal body tissues, but with LV output mainly distributed to the upper body and the bulk of RV output crossing the ductus arteriosus (a vascular channel connecting the pulmonary trunk directly to the descending thoracic aorta) to provide most of the blood flow to the lower fetal body and placenta. Only a minor portion of the RV output passes to the fetal lungs, which have a high vascular resistance and do not participate in respiratory gas exchange as they are filled with fluid. However, the RV is the functionally dominant heart chamber in the fetus as RV output exceeds LV output, while blood pressure in the pulmonary trunk is greater than in the aorta [16,20].

Substantial fetal growth and cardiopulmonary maturation occurs during late gestation (i.e. between the PT and NT time-points), manifested by rises in arterial blood pressures and ventricular outputs, as well as structural and functional maturation of the lungs [16,17]. Subsequently, the transition from fetal to newborn life at birth is characterized by, 1) a striking increase in blood flow to the lungs, which fill with air and become the site of gaseous exchange, 2) a rapid switchover to an LV functional dominance, as LV output increases to equal or even exceed RV output, while aortic blood pressure rises and pulmonary pressures fall, and 3) marked rises in whole body and myocardial oxygen consumption that are associated with substantial shifts in substrate utilization [16,17,19,20,37,38]. In addition, exposure to the extra-uterine environment also involves an expansion of the neonate’s microbiota [39].

Although pulmonary tissue miRNA expression changes have been reported with gestational age in mice, this has not been previously explored in fetal and neonatal sheep. For example, increases in murine pulmonary miR-146a and miR-27a [12] have been detected with advanced gestational age. Others have reported that in murine lungs miR-22-3p expression is increased concomitant to increased oxygen tension [40], whereas miR-210, is augmented in hypoxic lungs, pulmonary smooth muscle and other cell types exposed to hypoxic conditions [41,42]. In our study with NGS and quantitative RT PCR validation, neonatal lambs (consistent with an increase in blood oxygenation and high oxidative metabolic state) had increased pulmonary Bta-miR-22-3p_R+1, bta-miR-146a-R-2, and Hsa-miR-335* and decreased miR-210 compared to NT fetal lambs (in low oxygen state *in utero*). Compared to NT fetal lambs the pulmonary artery of neonates features a marked decrease in blood pressure. Pulmonary artery bta-miR-143_R-1 and hsa-miR-335* were decreased in neonates compared to NT in our study. Notably, it has been reported that a marked reduction in blood pressure is evident in miR-143/145 knock-out mice [43].

Consistent with the post-natal increase in oxidative metabolism and support of systemic cardiac output, the key changes in miR expression validated by quantitative RT-PCR were for Bta-miR-22-3p_R+1 and bta-miR-146a-3p_R+1 which increased and miR-210 which decreased in neonatal LV compared to NT fetuses. In contrast only miR-210 decreased in neonatal RV compared to NT fetuses. Although not previously reported in ovine cardiopulmonary tissue, and pending further specific validation and elaboration, these key identified shifts in Bta-miR-22-3p_R+1, bta-miR-146a-R-2, Hsa-miR-335* and miR-210, are in keeping with important processes occurring across the late gestation to neonatal period such as regulation of cell proliferation, cell differentiation, immune cell function and changes in cell hypoxia [44–46].

Our study detected moderate expression of miRNA-15 family members (miR-15a/b, miR-16, miR-195, miR-422, miR-497) in pulmonary, LV and RV tissues, however significant postnatal increases were not observed as reported previously in mice (postnatal day 10 versus postnatal day 1) [5]. Indeed the miRNA microarray study by Morrison et al. [18] also did not find a significant shift in miRNA-15 family that had been anticipated to correlate with miRNA-15-regulated cessation of cardiomyocyte proliferation in the ovine neonate as reported for previous mouse studies. This finding highlights that there are distinct specificities according to species, cell type and age that require detailed studies to be performed in large animal models and in human tissues.

In conclusion, our study has identified 1345 miRNAs in the fetal and neonatal ovine cardiopulmonary system and identified a narrow profile of specific miRNA that shift in expression occurring across the progression of late gestation to the neonatal period in LV and RV myocardium, main pulmonary artery and lungs. The ‘cardiopulmonary sheep miRNome’ we have compiled here is made available as a comparative resource for future studies of the gestational and postnatal ovine cardiopulmonary system. In addition to examining the functional roles of the presently identified miRNA shifts, future work is required to validate miRNA expression specifically for unique cell types isolated from cardiopulmonary tissues. Of particular importance, additional studies are required to determine the miRNA targets and regulatory significance of the relatively few miRNAs that alter in expression during the marked growth occurring in late gestation, the birth transition *per se* and post-natal growth to maturity.

## Supporting information

S1 Table Ovine miRNome. (XLSX)

S2 Table Tissue specific miRNA shifts by age group. (XLSX)
